# The Effect of Creatine Monohydrate on Mental Disorders: A Systematic Review of Randomized Controlled Trials: Effet du monohydrate de créatine sur les troubles mentaux : examen systématique des essais contrôlés à répartition aléatoire

**DOI:** 10.1177/07067437251408171

**Published:** 2026-01-20

**Authors:** Bassam Jeryous Fares, Carl Zhou, Nicholas Fabiano, Stanley Wong, Brendon Stubbs, Risa Shorr, David Puder, Darren G. Candow, Sergej M. Ostojic, Marco Solmi

**Affiliations:** 1 Faculty of Medicine, 12365 University of Ottawa, Ottawa, Canada; 2 SCIENCES Lab, Department of Psychiatry, 6363 University of Ottawa, Ottawa, Canada; 3 Department of Psychiatry, University of Toronto, Toronto, Canada; 4 Department of Psychological Medicine, 4616 Institute of Psychiatry, Psychology and Neuroscience, King's College London, London, UK; 5 Center for Sport Science and University Sports, University of Vienna, Wien, Austria; 6 Learning Services, 27337 The Ottawa Hospital, Ottawa, Canada; 7 12221 Mental Health Education & Research, Winter Park, Florida, USA; 8 Faculty of Kinesiology and Health Studies, 154143 University of Regina, Regina, Canada; 9 Department of Nutrition, 158919 University of Agder, Kristiansand, Norway; 10 Department of Child and Adolescent Psychiatry, Charité Universitätsmedizin, Berlin, Germany; 11 Department of Mental Health, The Ottawa Hospital, Ottawa, Canada; 12 Ottawa Hospital Research Institute: Clinical Epidemiology Program, 12365 University of Ottawa, Ottawa, Canada; 13 School of Epidemiology and Public Health, Faculty of Medicine, 12365 University of Ottawa, Ottawa, Canada

**Keywords:** creatine, bioenergetic, mental health, depression, bipolar, augmentation, créatine, bioénergétique, santé mentale, dépression, bipolaire, intensification

## Abstract

**Objective:**

The objective of this systematic review is to synthesize and evaluate the evidence involving creatine monohydrate supplementation (CrM) across mental disorders.

**Methods:**

MEDLINE, Embase, Cochrane, and PsycINFO were searched up to 09/30/2025 for randomized controlled trials (RCTs) investigating the effect of CrM on psychiatric symptoms and safety in participants with a mental disorder. Risk of bias was assessed.

**Results:**

Six articles from five RCTs were included (CrM: n = 126, placebo: n = 112; mean age=36 ± 14 years; male sex = 26%). Four RCTs reported on major depressive disorder (MDD), one bipolar depression. No other mental disorders were investigated. Two RCTs were low risk of bias and three had some concerns. CrM dosing ranged from 2 to 10 g/day for 4–8 weeks as adjunct treatment. In the treatment of MDD, CrM was tested as combination with escitalopram (k = 1, outperforming selective serotonin reuptake inhibitor (SSRI) + placebo; Cohen's d = 1.13 at 8 weeks), pharmacotherapy augmentation in adults (k = 1) and female adolescents (k = 1, no difference vs placebo), psychotherapy augmentation (k = 1, cognitive behavioural therapy (CBT) + CrM outperforming CBT + placebo) in MDD, and as pharmacotherapy augmentation in bipolar depression (k = 1, no difference vs placebo augmentation). Two trials in MDD found a correlation between CrM brain N-acetylaspartate and phosphocreatine, which was associated with larger improvement. CrM was generally well-tolerated. Two CrM out of 17 participants experienced hypomania/mania.

**Conclusion:**

CrM shows promise as a combination treatment with SSRIs or for augmenting psychotherapy in MDD in adults. Double-blind, large-scale RCTs investigating the efficacy of CrM, with and without first-line therapies, are needed across mental disorders.

## Résumé

**Objectif:**

Cet examen systématique vise à synthétiser et à évaluer les données probantes portant sur le monohydrate de créatine relativement à l’ensemble des troubles mentaux.

**Méthodes:**

Des recherches ont été effectuées sur MEDLINE, Embase, Cochrane et PsycINFO jusqu’au 30 septembre 2025 pour trouver des essais contrôlés à répartition aléatoire (ECRA) portant sur l’effet du monohydrate de créatine sur les symptômes psychiatriques et son innocuité chez les participants atteints d’un trouble mental. Le risque de biais a été évalué.

**Résultats:**

Six articles tirés de cinq ECRA ont été examinés (monohydrate de créatine : n = 126, placebo : n = 112; âge moyen = 36 ± 14 ans; sexe masculin = 26%). Quatre ECRA ont rapporté des données sur le trouble dépressif majeur (TDM) et une, sur la dépression bipolaire. Aucun autre trouble mental n’a été étudié. Deux ECRA présentaient un faible risque de biais et trois suscitaient des préoccupations. La dose de monohydrate de créatine variait de 2 à 10 g/jour pendant 4 à 8 semaines comme traitement d’appoint. Dans le traitement du TDM, le monohydrate de créatine a été évalué en association avec l’escitalopram (k = 1, supériorité à une association ISRS + placebo; d = 1,13 à 8 semaines), dans un contexte d’intensification de la pharmacothérapie chez les adultes (k = 1) et les adolescentes (k = 1, aucune différence par rapport au placebo), dans un contexte d’intensification de la psychothérapie (k = 1, association TCC + monohydrate de créatine supérieure à l’association TCC + placebo) dans les cas de TDM et dans un contexte d’augmentation de la pharmacothérapie dans les cas de dépression bipolaire (k = 1, aucune différence par rapport à l’intensification du traitement placebo). Deux essais sur le TDM ont révélé une corrélation entre le monohydrate de créatine et la concentration d’acide N-acétylaspartique et de phosphocréatinine dans le cerveau, qui a été associée à une amélioration accrue. Le monohydrate de créatine a été généralement bien toléré. Deux participants sur dix-sept ayant reçu du monohydrate de créatine ont présenté une hypomanie/manie.

**Conclusion:**

Le monohydrate de créatine semble prometteur comme traitement d’association avec les ISRS ou pour intensifier la psychothérapie chez les adultes atteints de TDM. Des ECRA à double insu et à grande échelle devront être menés pour étudier l’efficacité du monohydrate de créatine avec et sans traitement de première intention contre l’ensemble des troubles mentaux.

## Introduction

Psychiatric disorders, such as mood disorders, schizophrenia, and anxiety disorders, are prevalent and challenging conditions that are linked to significant functional impairment and reduced quality of life.^1,2^ Despite the availability of various pharmacological treatments, many patients experience suboptimal therapeutic responses or partial remission of symptoms.^3^ Further, patients with psychiatric disorders may struggle with medication adherence secondary to intolerable adverse effects.^4^

The shortcomings of pharmacological treatments have prompted the exploration of alternative or adjunctive treatments, including dietary supplements, to improve therapeutic outcomes for individuals with mental disorders.^5–8^ A promising candidate is creatine, a nitrogen-containing compound that plays a crucial role in cellular energy metabolism, particularly in tissues with high-energy demands, such as the brain.^9,10^

Creatine is endogenously synthesized from reactions involving the amino acids arginine, glycine, and methionine in the brain. It is also obtained exogenously through dietary sources such as meat and fish.^9^ Creatine acts as a temporal energy buffer during energy-demanding processes, such as cerebral function.^10^ When energy demands are high, creatine phosphate donates its phosphate group to adenosine diphosphate (ADP) to regenerate adenosine triphosphate (ATP).^10^ Furthermore, owing to its relatively fast diffusion rate, creatine facilitates intracellular energy transport by helping shuttle high-energy phosphate groups from the mitochondria to areas of the cell where ATP is rapidly consumed. Creatine also exhibits anti-inflammatory and anti-oxidative effects.^11–13^ Creatine reduces markers of inflammation and creates a favourable environment for muscle growth.^12^ It also protects mitochondrial DNA, creatine kinase, and RNA from oxidative damage.^11,13^

Beyond its well-documented benefits for enhancing physical performance,^14^ there is growing interest in creatine's potential therapeutic value for brain health and psychiatric disorders.^15–17^ People with mental health disorders demonstrate poorer dietary habits with higher intake of processed food.^18,19^ These dietary patterns may lead to insufficient intake of creatine exogenously in individuals with mental health conditions. Emerging research suggests that creatine monohydrate supplementation (CrM) may offer some neuroprotective effects and therapeutic benefits for psychiatric disorders characterized by impaired brain energy metabolism and mitochondrial dysfunction. For instance, alterations in creatine metabolism have been observed in psychiatric conditions such as schizophrenia, major depressive disorder (MDD), and bipolar disorder.^20^ Furthermore, creatine concentrations in the prefrontal cortex are negatively correlated with depressive symptoms.^21^ Similarly, lower creatine concentrations in the left dorsolateral prefrontal cortex are associated with social anxiety disorder.^22^ Notably, a significant inverse association between dietary creatine intake and depression was observed in a nationally representative cohort of U.S. adults,^23^ suggesting that creatine may exert antidepressant-like effects. These findings have prompted further investigation into the potential role of creatine for treating mental disorders.

Previous studies have explored the potential of creatine as an adjunctive treatment for mental disorders, particularly in cases where conventional treatments, such as antidepressants and antipsychotics, showed limited efficacy.^17,24^ These studies suggested that creatine may enhance cognitive function, reduce depressive symptoms, and improve overall brain bioenergetics. However, the evidence remains mixed, with some studies reporting significant improvements in mood and cognition^17,24,25^ and others reporting minimal or no effects.^26,27^ These discrepancies highlight the need for a systematic review to summarize the existing literature and provide a clearer understanding of creatine's efficacy in treating mental disorders.

The objective of this systematic review is to evaluate the effect of CrM on various mental disorders as reported by randomized controlled trials (RCTs). Tolerability and safety of creatine are secondary outcomes. By consolidating the available evidence, this study aims to determine whether creatine holds promise as a viable adjunctive treatment in psychiatric care.

## Methods

This systematic review adhered to the Preferred Reporting Items for Systematic Reviews and Meta-Analyses 2020 guidelines (eTable 1).^28^ The protocol was uploaded to Open Science Framework *a priori* and can be found at https://osf.io/gyrx2/.

### Search Strategy and Inclusion Criteria

We systematically searched on September 30, 2025, MEDLINE, EMBASE, Cochrane, and PsycINFO for RCTs investigating the effect of creatine on psychiatric symptoms in individuals with an established mental disorder diagnosis, regardless of adjuvant treatment. A search strategy using search terms related to creatine and mental disorders was developed with the guidance of a health sciences librarian (RS) (eTable 2). To ensure comprehensive coverage, we also conducted manual searches of Google Scholar and ClinicalTrials.gov using the search terms. The reference lists of the included studies were also screened to capture additional relevant articles. Studies were included if they met the following criteria: (1) RCT design, (2) include participants with mental disorders that were classified using Diagnostic and Statistical Manual of Mental disorders or International Classification of Diseases criteria, (3) investigate the effect of creatine on psychiatric symptoms as measured by a standardized scale, regardless of other adjuvant/underlying treatment received by participants.

### Study Screening

Eligible studies were imported into Covidence^29^ through which abstract and full-text screening were conducted. Both title and full-text screenings were conducted in duplicate by two independent reviewers (BJ, CZ, NF, SW). Discrepancies were resolved through consensus mediated by a third author.

### Data Extraction

Two reviewers (BJ, CZ) independently extracted relevant data from included articles onto a Microsoft Excel spreadsheet designed *a priori*. For each included trial, the following information was collected: trial identifiers, first author, publication year, publication DOI, country, inclusion/exclusion criteria, blinding, trial arms, participant demographics (age, sex, race), diagnosis, intervention details (including dosage, sample size, control group), augmentation/underlying treatment details, follow-up duration, scales for efficacy domain, primary and secondary efficacy outcome scale at baseline/endpoint/change values in both groups, number of completers in each group, and adverse events (AEs) as reported by the authors. Discrepancies were discussed and resolved by consensus mediated by a third author.

### Outcomes

Primary outcomes reported were disease-specific mental health symptoms (e.g., depressive symptoms in MDD) as demonstrated by clinical scales and remission rates from psychiatric diagnoses following treatments. Secondary outcomes included AEs, trial discontinuation rates, cognitive measures, and data on brain bioenergetics as demonstrated by spectroscopy and brain imaging studies.

### Risk of Bias

The Cochrane's Risk of Bias 2 tool^30^ was used to assess the risk of bias of all studies by two independent authors (BJ, CZ). For each domain, one of the following three judgments was assigned: “high-risk,” “low-risk,” or “some concerns.” Consensus mediated by a third author resolved any discrepancies.

### Data Analysis

Due to substantial heterogeneity in trial populations, mental disorders, interventions/controls, and outcome measures, a meta-analysis was not feasible. Instead, we narratively summarized the findings from the included trials.

## Results

### Search Results, Baseline, and Design Characteristics of Included Trials

**Figure 1.**
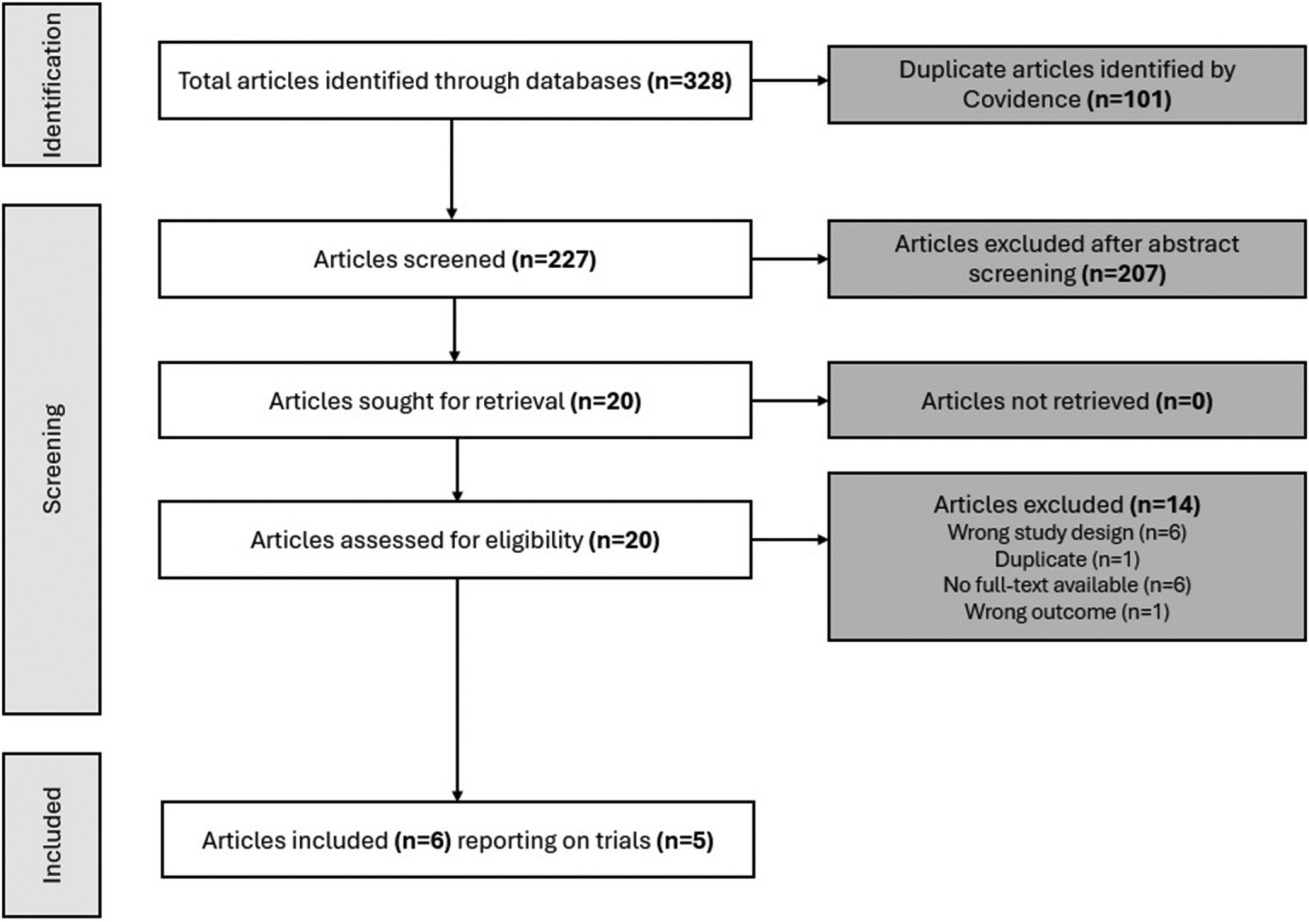
PRISMA study selection flow diagram. PRISMA=Preferred Reporting Items for Systematic Reviews and Meta-Analyses.

Initially, 328 records were retrieved from searches; 101 duplicates were removed, and 227 unique records were identified, with 207 excluded after title and abstract screening (Figure 1). The full texts of the remaining 20 articles were reviewed, six of which were included in this review, with the remaining 14 excluded (eTable 3). The six studies reported data from five RCTs.^17,25,27^^31–33^ The trials were conducted in South Korea (n = 1),^17,33^ USA (n = 1),^25^ Brazil (n = 1),^32^ Israel (n = 1),^27^ and India (n = 1).^31^ The trials included 238 baseline participants (CrM: n = 126, placebo: n = 112), with a mean age of 36 ± 14 years; 26.0% of participants were males. Only one trial reported data on race and ethnicity, with white and African American participants comprising 97.1% and 2.9% of the sample, respectively.^25^

The majority of the trials reported on MDD,^17,25,27,31,33^ with one trial reporting on bipolar disorder patients who were currently experiencing a major depressive episode.^32^ No trials reporting on other mental health conditions were found. Across the trials, the dosing of CrM and follow-up periods ranged from 2 g to 10 g daily and 4 weeks to 8 weeks, respectively (Table 1). One trial administered a CrM and pharmacotherapy combination to treat MDD in a sample of patients who were medication-free for at least 8 weeks at the time of enrolment.^17,33^ One trial augmented MDD pharmacotherapy with CrM in patients without signs of clinical improvement after at least 3 weeks on pharmacotherapy.^27^ Similarly, a trial assessed the efficacy of CrM in treating MDD in adolescent females with ongoing pharmacotherapy for at least 8 weeks.^25^ One trial assessed the efficacy of CrM in treating MDD in combination with cognitive behavioural therapy (CBT) in patients without pharmacotherapy treatment for at least 8 weeks before the start of the trial.^27^ Another trial assessed the efficacy of CrM augmentation to patients’ established pharmacotherapy regimen for bipolar disorder I or II with a current major depressive episode.^32^

**Table 1.** Baseline Characteristics of the Included Studies (n = 6).

Author, year	Double- blind	Mental disorder	Mean age (SD)	Male (%)	Intervention	Control	Duration	Augmented/ combination treatment
Sherpa et al. 2024	Yes	MDD	30.4 (7.4)	50	Creatine Monohydrate (5 g daily; n = 50)	Placebo once daily (n = 50)	8 weeks	Combination with biweekly individual CBT
Nemets & Levine 2013	Yes	MDD	55.4 (12.7)	22.2	Creatine Monohydrate (5 g daily; n = 5, or 10 g daily; n = 4)	Placebo once daily (n = 9)	4 weeks	Augmentation to patient's established medication regimen
Toniolo et al. 2018	Yes	Bipolar I disorder and bipolar II disorder	43.9 (9.2)	25.9	Creatine Monohydrate (6 g daily; n = 16)	Placebo once daily (n = 11)	6 weeks	Augmentation to patient's established medication regimen
Kondo et al. 2016	Yes	MDD	17.1 (2.1)	0	Creatine Monohydrate (2 g daily; n = 9, 4 g daily; n = 8, 10 g daily; n = 8)	Placebo once daily (n = 8)	8 weeks	Augmentation to SSRI treatment for ≥ 8 weeks at a dosage equivalent to ≥ 40 mg/day fluoxetine
Yoon et al. 2016	Yes	MDD	45.5 (11.6)	0	Creatine Monohydrate (5 g daily; n = 17)	Placebo once daily (n = 17)	8 weeks	Combination with SSRI. Initial dose of 10 mg daily of escitalopram for week 1, with subsequent increase in dosing up to 20 mg daily according to the clinician's judgement
Lyoo et al. 2012	Yes	MDD	46.6 (11.2)	0	Creatine Monohydrate (5 g daily; n = 25)	Placebo once daily (n = 27)	8 weeks	Combination with SSRI. Initial dose of 10 mg daily of escitalopram for week 1, with subsequent increase in dosing up to 20 mg daily according to the clinician's judgement

SD = standard deviation. CBT=cognitive behavioural therapy. SSRI = Selective serotonin reuptake inhibitor; MDD=major depressive disorder.

### Major Depressive Disorder

In two studies from the same RCT, Lyoo et al.^17^ (n = 52) and Yoon et al.^33^ (n = 34) examined the effects of CrM in combination (started concurrently) with pharmacotherapy on depressive symptoms in women (NCT00729755) with an age range of 19–65 years. The participants had a diagnosis of MDD with a current major depressive episode. Participants in the intervention group were treated with 3 g/day of CrM for the first week, followed by 5 g/day for 7 weeks. Trial participants were prescribed doses of escitalopram of 10 mg/day for the first week, followed by 20 mg/day for the remaining 7 weeks. Capsules containing dextrin, identical in appearance to capsules containing CrM, were given to the placebo group. While both groups in Lyoo et al.^17^ experienced reductions in depressive symptoms throughout the trial, individuals on CrM reported larger depressive symptom improvement (p < 0.001), as demonstrated by the HAM-D (CrM=5.4 ± 3.0, placebo=9.8 ± 3.5; Cohen's d = 1.13), Montgomery-Åsberg Depression Rating Scale (MADRS; z = -3.55), and Clinical Global Impression severity subscale (CGI-S; z = -3.89). With remission defined as a HAM-D score <8, 52% of participants on CrM achieved remission at week 8 compared with 25.9% of the participants on placebo and escitalopram (p = 0.008).^17^ Further, using proton magnetic resonance spectroscopy, Yoon et al.^33^ demonstrated a negative correlation between depressive symptoms and prefrontal N-acetylaspartate, a marker for mitochondrial function and neuronal viability,^34^ in women with MDD (Spearman rho=-0.49, p = 0.004). Prefrontal N-acetylaspartate increased significantly in patients receiving CrM and escitalopram compared with patients receiving placebo and escitalopram after 8 weeks of treatment (z = 2.47, p = .01, effect size = 0.73).

In a sample of patients with major depression (n = 18), Nemets & Levine^27^ examined the effect of different doses of CrM on patients’ HAM-D scores (mean age (standard deviation (SD)) = 55.4 (12.7)). The authors investigated CrM in patients who had not responded to antidepressant therapy for a minimum of 3 weeks, as demonstrated by less than a 50% decrease from baseline HAM-D score. Patients either received 3 g of CrM/day for the first week and 5 g/day for another 3 weeks, 5 g of CrM/day for the first week followed by 10 g/day for the remaining 3 weeks, or placebo doses equivalent to CrM. Patients’ pre-existing antidepressant treatment was not changed during the trial participation. Patients’ antidepressant treatments included selective serotonin reuptake inhibitors (SSRIs), serotonin norepinephrine reuptake inhibitors, and norepinephrine and specific serotonergic antidepressants. Benzodiazepines were allowed, not exceeding a lorazepam equivalent dose of 1–2 mg daily. While all groups exhibited improvement in their HAM-D scores by week 4, there was no significant treatment or dose effect on HAM-D scores across groups (CrM 5g = 13.4 ± 8.8, placebo 5g = 14.6 ± 13.0, CrM 10g = 14.3 ± 10.5, placebo 10g = 16.3 ± 14.8).

Similarly, Kondo et al.^25^ assessed the impact of different CrM doses in a sample of females with a primary diagnosis of MDD (n = 33). However, unlike Lyoo et al.,^17^ Yoon et al.,^33^ and Nemets & Levine,^27^ their sample consisted mainly of adolescents with a mean age of 17 ± 2 years as opposed to adults. The patients had been treated, at the time of enrolment, with an SSRI for ≥8 weeks at a dosage of ≥40 mg/day fluoxetine or its equivalent in other SSRI treatments (i.e., escitalopram, sertraline, and citalopram). The participants had a depressive episode with moderate severity as evidenced by their scores on CGI-S (≥4), MADRS (>25), and Children's Depression Rating Scale-Revised (CDRS-R; >40). The participants in the trial were randomized to placebo or a dose of 2 g, 4 g, or 10 g of CrM/day for 8 weeks. There were no significant differences between the different dosing groups or with placebo on their CDRS-R scores at the end of the trial (CrM 2g = 34.8 ± 8.20, CrM 4g = 41.8 ± 14.78, CrM 10g = 36.1 ± 13.96, placebo=43.0 ± 12.58). Importantly, the group who consumed 10 g/day of CrM increased their frontal lobe phosphocreatine (PCr) concentration by 9.1% compared to 4.1% and 4.6% for the 2 g/day and 4 g/day CrM groups, respectively. Regression analyses showed that prefrontal PCr levels were inversely correlated with depression scores (p = 0.02).

Unlike the other trials, Sherpa et al.^31^ investigated the treatment of depression with CrM (5 g/day for 8 weeks) in combination (started concurrently) with psychotherapy (CBT). Recruited participants were young and middle-aged adults (mean age (SD) = 30.4 (7.4)) with a diagnosis of MDD with a current depressive episode (n = 100). Participants had discontinued any pharmacotherapy for at least 8 weeks before the screening evaluation. All participants received individual CBT once every 2 weeks with a clinical psychologist. Participants receiving combination CBT and CrM reported lower scores on the Patient Health Questionnaire 9 compared with those receiving CBT and placebo (oral starch) by the end of the trial (CBT + CrM=5.8 ± 4.8, CBT + placebo=11.9 ± 6.6; p < 0.05), despite similar baseline scores.

### Bipolar Disorder

Only one trial examined CrM in the context of bipolar disorder.^32^ The trial included young and middle-aged adults (mean age (SD) = 43.9 (9.2)) with a diagnosis of bipolar disorder (I or II) with a current depressive episode, with a score of 20 or greater on the MADRS (n = 27). To be included, participants had to have a stable dose of antipsychotics or mood stabilizers for at least 2 weeks and/or antidepressants for at least 4 weeks. Participants were randomized to 6 g of CrM or 6 g of corn starch per day for 6 weeks. There were no significant differences between the two groups with regard to changes in scores throughout the trial on the MADRS, HAM-D, CGI-S, Young Mania Rating Scale, and the Functioning Assessment Short Test.

### All-Cause and Side Effect-Related Discontinuation

Generally, there were no significant differences in withdrawal rate across trials in relation to CrM. Nemets & Levine^27^ reported no participant withdrawal or loss to follow-up during the trial. There was no significant difference in withdrawal rate in Toniolo et al.^32^ between the CrM (n = 5; 29.4%) and placebo (n = 7; 38.9%) groups; however, two patients in the CrM arm experienced hypomania/mania, and two had increased depressive symptoms. Three participants in the CrM group (12%) in Kondo et al.^25^ withdrew compared with two participants in the placebo group (25%). It was not reported if the difference in withdrawal rate was significant, and none of the withdrawals were due to AEs. There was a withdrawal rate of 40% (n = 20) each in both the CrM and placebo groups in Sherpa et al.,^31^ with nine (18%) and eight (16%) participants in the CrM and placebo groups discontinuing the trial due to AEs, respectively. Lyoo et al.^17^ reported no significant difference in withdrawal rate between the CrM (n = 8; 32.0%) and placebo groups (n = 5; 18.5%), with two and one of the participants in the CrM and placebo groups, respectively, withdrawing as a consequence of AEs. In a subsample of the same RCT as Lyoo et al.,^17^ there was no significant difference in withdrawal rate in Yoon et al.^33^ between the CrM (n = 6; 35.3%) and placebo (n = 1; 0.06%) groups.

### Safety Outcomes

Across all trials, participants receiving CrM exhibited some AEs, including pruritus, nausea, vomiting, diarrhoea, constipation, headache, abdominal pain, weight gain, dizziness, appetite changes, and difficulties with sleeping. More participants in the placebo + CBT group in Sherpa et al.^31^ reported at least one AE (n = 34; 68%) compared to CrM + CBT (n = 30; 60%); however, it is not reported whether or not the difference between groups is significant. Similarly, placebo participants reported relatively more gastrointestinal side effects in Kondo et al.^25^ (n = 4, 67%) compared to participants receiving CrM (n = 11, 50%), but the significance of the difference was not reported. There were no significant differences in AE prevalence in Lyoo et al.^17^ between placebo (n = 43.4%) and CrM (n = 46.4%) groups. None of the participants in the intervention or control groups in Nemets & Levine^27^ reported clinically relevant AEs.

With regard to specific AEs, Lyoo et al.^17^ reported no significant differences in the prevalence of tension headaches, nausea and vomiting, sleep difficulties, dizziness, agitation, decreased appetite, increased sweating, and difficulty in concentration between CrM and placebo groups. Likewise, there were no significant differences reported by Toniolo et al.^32^ in the frequency of pruritus, cramps, constipation, diarrhoea, limb oedema, nausea, vomiting, reflux, somnolence, dizziness, asthenia, and abdominal pain. However, participants receiving the placebo treatment reported more tension headaches (n = 6; 54.5%) compared to participants receiving CrM (n = 2; 12.5%), and two patients who received CrM experienced a hypomanic/manic switch by the end of week 2 of the trial. Further, Sherpa et al.^31^ also reported the prevalence of specific AEs in their sample, but it is not reported whether or not the differences in prevalence between groups are significant. Serum creatinine was also assessed in two trials.^17,25^ Kondo et al.^25^ reported no significant differences in serum creatinine concentration between the placebo (0.79 ± 0.051 mg/dL) and CrM groups (CrM 2g = 0.84 ± 0.062 mg/dL, CrM 4 g = 0.74 ± 0.057 mg/dL, CrM 10 g = 0.88 ± 0.053 mg/dL) at the end of the trial. On the other hand, Lyoo et al.^17^ found elevated serum creatinine in the CrM group (0.79 ± 0.15 mg/dL) at the end of the trial compared to the placebo group (0.67 ± 0.11 mg/dL), but the concentrations remained within the normal range.

### Risk of Bias

The included trials were determined to be at low risk of bias (n = 2) and some concerns with bias (n = 3; Table 2). The primary areas of concern regarding bias relate to the randomization process (n = 2) and missing outcome data (n = 2). Two trials reported baseline differences between the intervention and control groups. Furthermore, there was missing outcome data in two separate trials due to participant loss to follow-up (eFigure 1).

**Table 2.**
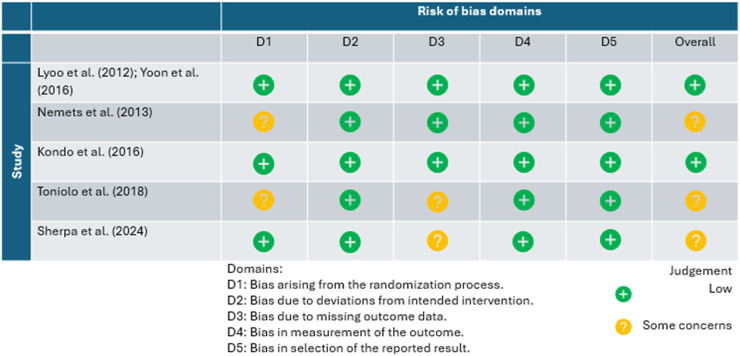
Risk of Bias Assessment Using Cochrane Risk of Bias Tool 2.

## Discussion

To the best of our knowledge, this is the first systematic review to investigate the potential benefits and safety of CrM in adults with mental disorders. The majority (n = 4) of trials focused on unipolar depression,^17,25,27^^31–33^ with one trial reporting on bipolar depression.^32^ The RCTs ranged in length from 4 to 8 weeks, with creatine dosage between 2 g to 10 g daily. Creatine augmented or was combined with pharmacotherapy in the majority (n = 4) of trials,^17,25,27,32,33^ with one recent RCT using CrM as an augment to CBT.^31^ In general, creatine led to improved antidepressant response at trial endpoints.^17^ There were no significant differences regarding acceptability, serious/severe AEs; however, mild AEs such as gastrointestinal distress were reported. Further, in a small trial for bipolar depression, two patients on CrM developed hypomania/mania early in the trial, warranting further investigation. This paper serves as the first systematic synthesis of CrM as a treatment for mental disorders.

These findings align with a previous non-systematic review of clinical trials (not exclusively RCTs) involving CrM for the treatment of depression, which found CrM to be well-tolerated with a noticeable antidepressant effect.^35^ Although there is a paucity of RCTs for other mental disorders, small open-label trials for schizophrenia^36^ and PTSD^16^ have yielded positive results. For schizophrenia, seven inpatients had their treatment augmented with 10 g of CrM per day for 6 months, which demonstrated improvements in schizophrenia symptomatology, ward behaviours, and tardive parkinsonism; however, no measurable change in cognition.^36^ For PTSD, 10 outpatients with treatment-resistant PTSD were augmented with 5 g of CrM per day for 4 weeks, which resulted in beneficial effects on sleep, depressive, and PTSD symptomatology.^16^

The reasons why CrM may have a positive impact on depression are yet to be fully elucidated. However, since depression (both unipolar and bipolar) can be conceptualized through the lens of altered brain metabolism, this could be one mechanism.^37^ Depression represents a state of impaired energy metabolism, which involves mitochondrial pathology, including changes that affect oxidative phosphorylation; a process responsible for the generation of energy via ATP. The majority of ATP synthesis occurs during aerobic cellular respiration, where ATP is formed from ADP using energy from the mitochondrial proton gradient; however, this process is complex and time-consuming. Here, creatine, stored as PCr in the brain, can be used to quickly replenish ATP levels in the brain (by donating a phosphoryl group to ADP) when there are significant ongoing metabolic demands, such as during depression. However, when exposed to prolonged metabolic stress (i.e., a prolonged depressive episode), brain PCr stores can also become depleted forcing cells to rely on the less efficient glycolytic pathway, potentially leading to mitochondrial dysfunction, increased oxidative stress and altered brain metabolism.^15,37^ In fact, previous research has identified an association between low creatine levels in the prefrontal cortex and severity of depressive symptoms.^21^ Altered brain bioenergetics may underlie the predisposition to depression in individuals with chronic health conditions, such as diabetes and heart failure.^35^ The causal relationship; however, between mental health and brain bioenergetics remains unclear. In addition to its role in supporting brain metabolism, creatine may facilitate the synthesis, release, and reuptake of monoamines such as serotonin and dopamine,^38^ and also exert antioxidant effects, mitigate mitochondrial dysfunction, and promote neuronal survival.^11^ Creatine also acts as a neuromodulator and neurotransmitter, acting on the D_1_ and D_2_ dopamine receptors, serotonergic 5-HT _1A_ receptors, α_1_-adrenoceptors and adenosine A_1_ and A_2a_ receptors.^39^ Creatine may also exert electrophysiological effects in the brain through N-methyl-D-aspartate (NMDA) and calcineurin pathways. NMDA receptor antagonists attenuated the creatine induced increase in Na+,K + -ATPase activity and amplitude of neuronal population spikes in rat hippocampal slices.^40,41^ Further, there is some evidence that short-term (21 days) CrM increases brain-derived neurotrophic factor, a nerve growth factor involved in neuronal survival and plasticity and neurotransmitter release^42^ and upregulates expression of peroxisome proliferator-activated receptor gamma coactivator 1-alpha (PGC1 ***α***), the master regulator of mitochondrial biogenesis in mice^43^ and reduces serum measures of neurofilment light chain, biomarker indicator or neuronal damage in humans.^44^ It is interesting to note that individuals with suboptimal creatine intake are at an increased risk of experiencing sleep disturbances.^45^ Further, a single dose of creatine may help mitigate the effect of fatigue-induced cognitive deficits in sleep-deprived participants.^46^ Therefore, creatine may exert its influence on depressive and bipolar symptoms through the modulation of sleep patterns and behaviours.

Dietary intake, via red meat and fish, represents a significant source of creatine in humans. As such, those who adhere to vegetarian or vegan diets have been found to have 30% lower creatine content than their meat-eating counterparts.^47^ However, there were no differences in brain creatine in the posterior cingulate cortex between omnivores and vegetarians despite their creatine-poor diets.^48^ Despite this, at the population-level, and without excluding participants on vegan or vegetarian diets, a study of 22,692 U.S. adults found an inverse, stepwise association between dietary creatine intake and depression, with the strongest effect in females and those aged 20–39.^23^ Although this observational finding suggests a link between dietary creatine and depression, it is important to note that one would need to eat approximately 3 pounds of beef in order to get around 5 g of creatine.^37^ As such, dietary creatine levels and supplemental creatine levels (as seen in the included studies in this review) represent significantly different levels of creatine intake; as such, it is not recommended to use meat as one's primary source of creatine.

This systematic review has several strengths. First, this is the first systematic review assessing creatine as a treatment across mental disorders, using stringent methodology, and posing no restrictions on outcomes of interest. Second, reporting bias was minimized through the posting of an *a priori* protocol. Third, the search strategy was conducted across numerous databases, with screening and extraction done in duplicate. Fourth, we focused exclusively on RCTs, which represents the highest quality of evidence and allows temporality to be established between exposure and outcome while simultaneously addressing confounding through randomization. Fifth, half of included studies were deemed to be low risk of bias based on the ROB2, with none being high risk of bias, highlighting their rigorous methodology and reliable results.

Despite these strengths, several limitations must be noted. First, due to the significant heterogeneity of included studies, a meta-analysis was not possible and results were reported descriptively. Second, the majority of included studies focused on unipolar depression (with one study focusing on bipolar depression); however, there were no RCTs on other mental disorders available. Third, the short follow-up of 4 to 8 weeks did not allow for assessment of the durability of efficacy, safety, and acceptability that are necessary for mental disorders which often require long-term treatment. Fourth, many RCTs were proof-of-concept with small sample sizes, which were likely underpowered to detect significant changes in psychiatric symptoms. Fifth, although the creatine doses ranged from 2 g to 10 g daily, the majority of studies used ≤5 g daily; recent research has suggested that this dose may be too low to significantly change brain creatine concentrations, and by extension, expected changes in psychiatric symptoms.^37^ Sixth, the analysis was limited to a single chemical form of creatine – CrM – thereby restricting the generalizability of the findings to other creatine formulations.

These RCTs have identified a valuable signal for future creatine research, with particular areas of focus.^37^ First, due to the favourable risk-to-benefit ratio of creatine, RCTs should be conducted using creatine as an adjunctive treatment across mental disorders (beyond just depression), measuring various psychiatric outcomes. Second, RCTs with larger sample sizes and more diverse patient populations are needed to provide additional information on the efficacy, acceptability and safety in people with mental disorders. Third, in future studies, creatine should be paired with other primary treatment modalities, such as exercise, which has demonstrated equivalence in antidepressant effect among antidepressants and CBT for the treatment of depression.^49,50^ Fourth, higher doses of creatine ≥10 g daily for a longer period of time (>8 weeks) remain largely unexplored and may result in higher brain creatine concentrations, and in turn changes in psychiatric outcomes.^37^ In particular, a recent trial administered a high single dose of creatine (0.35 g/kilogram of bodyweight) among healthy, but sleep-deprived (a state of high metabolic demand – similar to depression) subjects, which partially reversed metabolic alterations and cognitive deterioration.^46^ Fifth, guanidinoacetic acid (GAA), a direct natural precursor to creatine, has demonstrated greater rise in brain creatine levels than creatine itself; therefore GAA supplemented alone or with creatine may be a promising strategy to maximize brain creatine levels.^51^

Overall, this systematic review narratively summarized five RCTs, which largely focused on unipolar depression (with one RCT on bipolar depression), using creatine as an augmentation to traditional treatment. In general, creatine led to an improved antidepressant response at trial endpoints, with indication of a more rapid response when combined with antidepressants. Creatine was well-tolerated, with mild gastrointestinal distress reported as a side effect, with one trial of bipolar depression experiencing transition to hypomania/mania, warranting further investigation. Future research should aim to investigate the full spectrum of mental disorders, larger sample sizes, higher doses (≥10 g daily) for a longer period of time (>8 weeks), GAA supplementation, and pairing creatine with other treatment modalities (i.e., exercise).

## Supplemental Material

sj-docx-1-cpa-10.1177_07067437251408171 - Supplemental material for The Effect of Creatine Monohydrate on Mental Disorders: A Systematic Review of Randomized Controlled Trials: Effet du monohydrate de créatine sur les troubles mentaux : examen systématique des essais contrôlés à répartition aléatoire Supplemental material, sj-docx-1-cpa-10.1177_07067437251408171 for The Effect of Creatine Monohydrate on Mental Disorders: A Systematic Review of Randomized Controlled Trials: Effet du monohydrate de créatine sur les troubles mentaux : examen systématique des essais contrôlés à répartition aléatoire by Bassam Jeryous Fares, Carl Zhou, Nicholas Fabiano, Stanley Wong, Brendon Stubbs, Risa Shorr, David Puder, Darren G. Candow, Sergej M. Ostojic and Marco Solmi in The Canadian Journal of Psychiatry
