# Supplementary material for: The Effect of Creatine Monohydrate on Mental Disorders: A Systematic Review of Randomized Controlled Trials: Effet du monohydrate de créatine sur les troubles mentaux : examen systématique des essais contrôlés à répartition aléatoire
Source: Can J Psychiatry. 2026 Jan 20:07067437251408171. Online ahead of print. doi: 10.1177/07067437251408171 (PMC12823350; doi:10.1177/07067437251408171)
Supplement: sj-docx-1-cpa-10.1177_07067437251408171 - Supplemental material for The Effect of Creatine Monohydrate on Mental Disorders: A Systematic Review of Randomized Controlled Trials: Effet du monohydrate de créatine sur les troubles mentaux : examen systématique des essais contrôlés à répartition aléatoir [file sj-docx-1-cpa-10.1177_07067437251408171.docx]

**Supplementary Material**

**The effect of creatine on mental disorders: a systematic review of randomized controlled trials**

Bassam Jeryous Fares^1^*, Carl Zhou^2^*, Nicholas Fabiano^2^*, Stanley Wong^2,3^*, Brendon Stubbs^4,5^, Risa Shorr^6^, David Puder MD^7^, Darren G. Candow PhD^8^, Marco Solmi^2,9-12^

1. Faculty of Medicine, University of Ottawa, Ottawa, Canada
2. SCIENCES Lab, Department of Psychiatry, University of Ottawa, Ottawa, Canada
3. Department of Psychiatry, University of Toronto, Toronto, Canada
4. Department of Psychological Medicine, Institute of Psychiatry, Psychology and Neuroscience, King’s College London, London, United Kingdom
5. Center for Sport Science and University Sports, University of Vienna, Wien, Austria
6. Learning Services, The Ottawa Hospital, Ottawa, Canada
7. Loma Linda University School of Medicine, Loma Linda, CA, USA
8. Faculty of Kinesiology and Health Studies, University of Regina, Regina, Canada
9. Department of Child and Adolescent Psychiatry, Charité Universitätsmedizin, Berlin, Germany
10. Department of Mental Health, The Ottawa Hospital, Ottawa, Canada
11. Ottawa Hospital Research Institute: Clinical Epidemiology Program, University of Ottawa, Ottawa, Canada
12. School of Epidemiology and Public Health, Faculty of Medicine, University of Ottawa, Ottawa, Canada

*Bassam Jeryous Fares, Carl Zhou, Nicholas Fabiano, and Stanley Wong contributed equally to this study and are co-first authors.

Marco Solmi (Corresponding author): msolmi@toh.ca

**Funding sources**: None

**Disclosures**: MS received honoraria/has been a consultant for AbbVie, Angelini, Boehringer Ingelheim, Lundbeck, and Otsuka. BS is on the Editorial Board of Ageing Research Reviews, Mental Health and Physical Activity, The Journal of Evidence Based Medicine, and The Brazilian Journal of Psychiatry. BS has received honorarium from a co-edited book on exercise and mental illness (Elsevier) and associated training courses, and unrelated advisory work from ASICS and FitXR LTD.

eTable 1. PRISMA 2020 Checklist.

| **Section and Topic** | **Item #** | **Checklist item** | **Location where item is reported** |
| --- | --- | --- | --- |
| **TITLE** | | |  |
| Title | 1 | Identify the report as a systematic review. | Title page |
| **ABSTRACT** | | |  |
| Abstract | 2 | See the PRISMA 2020 for Abstracts checklist. | Abstract |
| **INTRODUCTION** | | |  |
| Rationale | 3 | Describe the rationale for the review in the context of existing knowledge. | Introduction |
| Objectives | 4 | Provide an explicit statement of the objective(s) or question(s) the review addresses. | Introduction |
| **METHODS** | | |  |
| Eligibility criteria | 5 | Specify the inclusion and exclusion criteria for the review and how studies were grouped for the syntheses. | Methods, Search strategy and inclusion criteria |
| Information sources | 6 | Specify all databases, registers, websites, organisations, reference lists and other sources searched or consulted to identify studies. Specify the date when each source was last searched or consulted. | Methods, Search strategy and inclusion criteria |
| Search strategy | 7 | Present the full search strategies for all databases, registers and websites, including any filters and limits used. | eTable 2 |
| Selection process | 8 | Specify the methods used to decide whether a study met the inclusion criteria of the review, including how many reviewers screened each record and each report retrieved, whether they worked independently, and if applicable, details of automation tools used in the process. | Methods, Search strategy and inclusion criteria |
| Data collection process | 9 | Specify the methods used to collect data from reports, including how many reviewers collected data from each report, whether they worked independently, any processes for obtaining or confirming data from study investigators, and if applicable, details of automation tools used in the process. | Methods, Study screening, Data extraction |
| Data items | 10a | List and define all outcomes for which data were sought. Specify whether all results that were compatible with each outcome domain in each study were sought (e.g. for all measures, time points, analyses), and if not, the methods used to decide which results to collect. | Methods, Data extraction |
|  | 10b | List and define all other variables for which data were sought (e.g. participant and intervention characteristics, funding sources). Describe any assumptions made about any missing or unclear information. | Methods, Data extraction |
| Study risk of bias assessment | 11 | Specify the methods used to assess risk of bias in the included studies, including details of the tool(s) used, how many reviewers assessed each study and whether they worked independently, and if applicable, details of automation tools used in the process. | Methods, Risk of bias and certainty of evidence |
| Effect measures | 12 | Specify for each outcome the effect measure(s) (e.g. risk ratio, mean difference) used in the synthesis or presentation of results. | Methods, Statistical analysis |
| Synthesis methods | 13a | Describe the processes used to decide which studies were eligible for each synthesis (e.g. tabulating the study intervention characteristics and comparing against the planned groups for each synthesis (item #5)). | Methods, Statistical analysis |
|  | 13b | Describe any methods required to prepare the data for presentation or synthesis, such as handling of missing summary statistics, or data conversions. | Methods, Statistical analysis |
|  | 13c | Describe any methods used to tabulate or visually display results of individual studies and syntheses. | Methods, Statistical analysis |
|  | 13d | Describe any methods used to synthesize results and provide a rationale for the choice(s). If meta-analysis was performed, describe the model(s), method(s) to identify the presence and extent of statistical heterogeneity, and software package(s) used. | Methods, Statistical analysis |
|  | 13e | Describe any methods used to explore possible causes of heterogeneity among study results (e.g. subgroup analysis, meta-regression). | Methods, Statistical analysis |
|  | 13f | Describe any sensitivity analyses conducted to assess robustness of the synthesized results. | Methods, Statistical analysis |
| Reporting bias assessment | 14 | Describe any methods used to assess risk of bias due to missing results in a synthesis (arising from reporting biases). | Methods, Risk of bias and certainty of evidence |
| Certainty assessment | 15 | Describe any methods used to assess certainty (or confidence) in the body of evidence for an outcome. | Methods, Risk of bias and certainty of evidence |
| **RESULTS** | | |  |
| Study selection | 16a | Describe the results of the search and selection process, from the number of records identified in the search to the number of studies included in the review, ideally using a flow diagram. | Results, Search results, baseline, and design characteristics of included trials |
|  | 16b | Cite studies that might appear to meet the inclusion criteria, but which were excluded, and explain why they were excluded. | Results, Search results, baseline, and design characteristics of included trials |
| Study characteristics | 17 | Cite each included study and present its characteristics. | Results, Search results, baseline, and design characteristics of included trials |
| Risk of bias in studies | 18 | Present assessments of risk of bias for each included study. | Results, Risk of bias and certainty of evidence |
| Results of individual studies | 19 | For all outcomes, present, for each study: (a) summary statistics for each group (where appropriate) and (b) an effect estimate and its precision (e.g. confidence/credible interval), ideally using structured tables or plots. | Results |
| Results of syntheses | 20a | For each synthesis, briefly summarise the characteristics and risk of bias among contributing studies. | Results, Risk of bias and certainty of evidence |
|  | 20b | Present results of all statistical syntheses conducted. If meta-analysis was done, present for each the summary estimate and its precision (e.g. confidence/credible interval) and measures of statistical heterogeneity. If comparing groups, describe the direction of the effect. | Results |
|  | 20c | Present results of all investigations of possible causes of heterogeneity among study results. | Results |
|  | 20d | Present results of all sensitivity analyses conducted to assess the robustness of the synthesized results. | Results |
| Reporting biases | 21 | Present assessments of risk of bias due to missing results (arising from reporting biases) for each synthesis assessed. | Results, Publication bias |
| Certainty of evidence | 22 | Present assessments of certainty (or confidence) in the body of evidence for each outcome assessed. | Results, Risk of bias and certainty of evidence |
| **DISCUSSION** | | |  |
| Discussion | 23a | Provide a general interpretation of the results in the context of other evidence. | Discussion |
|  | 23b | Discuss any limitations of the evidence included in the review. | Discussion |
|  | 23c | Discuss any limitations of the review processes used. | Discussion |
|  | 23d | Discuss implications of the results for practice, policy, and future research. | Discussion |
| **OTHER INFORMATION** | | |  |
| Registration and protocol | 24a | Provide registration information for the review, including register name and registration number, or state that the review was not registered. | Methods |
|  | 24b | Indicate where the review protocol can be accessed, or state that a protocol was not prepared. | Methods |
|  | 24c | Describe and explain any amendments to information provided at registration or in the protocol. | N/A |
| Support | 25 | Describe sources of financial or non-financial support for the review, and the role of the funders or sponsors in the review. | Title page |
| Competing interests | 26 | Declare any competing interests of review authors. | Title page |
| Availability of data, code and other materials | 27 | Report which of the following are publicly available and where they can be found: template data collection forms; data extracted from included studies; data used for all analyses; analytic code; any other materials used in the review. | Methods |

eTable 2. Search strategy.

| MEDLINE | EMBASE | Cochrane | PsycINFO |
| --- | --- | --- | --- |
| Ovid MEDLINE(R) ALL <1946 to September 30, 2025>  1 exp Mental Disorders/ 1516068  2 (mental disorder* or mental* ill* or mood disorder* or Neurocognitive Disorder*).tw,kf. 137161  3 (anxiety or psychotic or psychosis or psychoses or schizo* or bipolar or manic or mania or delirious or depressi* or depresse* or major depression* or obsessive-compulsive or obsessive compulsive or compulsive obsessive or OCD or agoraphob* or panic or phobia or phobic or melanchol* or neurosis or neurotic or neuroses or conversion disorder*).tw,kf. 1008280  4 (alzheimer* or dementia or "substance use disorder*" or substance abuse or drug abuse or opiate abuse or opiate addiction or opioid abuse or opioid addiction or alcohol abuse or alcoholism or Dissociative disorder* or Elimination Disorder* or impulse control disorder* or disruptive behavio?r* or dissocial disorder* or personality disorder* or ptsd or paraphilic disorder* or factitious disorder*).tw,kf. 480801  5 behavio?r disorder*.tw,kf. 8185  6 (autism or autistic or attention deficit or adhd).tw,kf. 115353  7 (eating disorder* or Anorexia or anorexic or bullimia or binge eating).tw,kf. 59917  8 gambling.tw,kf. 10215  9 post trauma*.tw,kf. 46139  10 Catatonia/ 2985  11 Catatoni*.tw,kf. 4236  12 neurodevelopment* disorder*.tw,kf. 20371  13 (psychiat* adj2 (illness* or disorder*)).tw,kf. 67801  14 or/1-13 2347976  15 creatine/ and Dietary Supplements/ 941  16 Creatine/ad, tu [Administration & Dosage, Therapeutic Use] 1077  17 (creatine adj5 (supplement* or loading)).tw,kf. 1606  18 ((exogenous or oral) adj3 creatine).tw,kf. 311  19 creatine intake.tw,kf. 57  20 (dietary creatine or creatine monohydrate).tw,kf. 631  21 or/15-20 2279  22 14 and 21 202  23 exp randomized controlled trial/ 626490  24 (random* or placebo).tw. 1655418  25 trial.tw. 835645  26 exp clinical trial/ 1008012  27 or/23-26 2389483  28 exp animals/ not humans/ 5273887  29 27 not 28 2201822  30 22 and 29 54 | Embase Classic+Embase <1947 to 2025 September 30>  1 exp mental disease/ 3057324  2 (mental disorder* or mental* ill* or mood disorder* or Neurocognitive Disorder*).tw. 168372  3 (anxiety or psychotic or psychosis or psychoses or schizo* or bipolar or manic or mania or delirious or depressi* or depresse* or major depression* or obsessive-compulsive or obsessive compulsive or compulsive obsessive or OCD or agoraphob* or panic or phobia or phobic or melanchol* or neurosis or neurotic or neuroses or conversion disorder*).tw. 1383551  4 (alzheimer* or dementia or "substance use disorder*" or substance abuse or drug abuse or opiate abuse or opiate addiction or opioid abuse or opioid addiction or alcohol abuse or alcoholism or Dissociative disorder* or Elimination Disorder* or impulse control disorder* or disruptive behavio?r* or dissocial disorder* or personality disorder* or ptsd or paraphilic disorder* or factitious disorder*).tw. 640682  5 behavio?r disorder*.tw. 11843  6 (autism or autistic or attention deficit or adhd).tw. 150856  7 (eating disorder* or Anorexia or anorexic or bullimia or binge eating).tw. 84622  8 gambling.tw. 12420  9 post trauma*.tw. 60569  10 Catatoni*.tw. 6770  11 neurodevelopment* disorder*.tw. 24878  12 (psychiat* adj2 (illness* or disorder*)).tw. 99092  13 exp "disorders of higher cerebral function"/ 1096931  14 or/1-13 3755267  15 creatine/ and (dietary supplement/ or diet supplementation/) 1551  16 creatine/ad, po [Drug Administration, Oral Drug Administration] 542  17 creatine phosphate/dt, iv, po 176  18 (creatine adj5 (supplement* or loading)).tw. 1923  19 ((exogenous or oral) adj3 creatine).tw. 378  20 creatine intake.tw. 66  21 (dietary creatine or creatine monohydrate).tw. 740  22 or/15-21 3209  23 14 and 22 464  24 exp clinical trial/ 1979872  25 exp controlled clinical trial/ 1052464  26 (random* or placebo or trial).tw. 2831994  27 double blind*.tw. 262810  28 24 or 25 or 26 or 27 3746558  29 (exp animals/ or nonhumans/ or animal experiments/) not exp humans/ 6129044  30 28 not 29 3484599  31 23 and 30 120 | EBM Reviews - Cochrane Central Register of Controlled Trials <September 2025>  1 exp Mental Disorders/ 108601  2 (mental disorder* or mental* ill* or mood disorder* or Neurocognitive Disorder*).tw,kw. 17280  3 (anxiety or psychotic or psychosis or psychoses or schizo* or bipolar or manic or mania or delirious or depressi* or depresse* or major depression* or obsessive-compulsive or obsessive compulsive or compulsive obsessive or OCD or agoraphob* or panic or phobia or phobic or melanchol* or neurosis or neurotic or neuroses or conversion disorder*).tw,kw. 179667  4 (alzheimer* or dementia or "substance use disorder*" or substance abuse or drug abuse or opiate abuse or opiate addiction or opioid abuse or opioid addiction or alcohol abuse or alcoholism or Dissociative disorder* or Elimination Disorder* or impulse control disorder* or disruptive behavio?r* or dissocial disorder* or personality disorder* or ptsd or paraphilic disorder* or factitious disorder*).tw,kw. 50640  5 behavio?r disorder*.tw,kw. 1848  6 (autism or autistic or attention deficit or adhd).tw,kw. 13018  7 (eating disorder* or Anorexia or anorexic or bullimia or binge eating).tw,kw. 8776  8 gambling.tw,kw. 948  9 post trauma*.tw,kw. 4814  10 Catatonia/ 26  11 Catatoni*.tw,kw. 144  12 neurodevelopment* disorder*.tw,kw. 680  13 (psychiat* adj2 (illness* or disorder*)).tw,kw. 5863  14 or/1-13 278348  15 creatine/ and Dietary Supplements/ 371  16 Creatine/ad, tu [Administration & Dosage, Therapeutic Use] 8  17 (creatine adj5 (supplement* or loading)).tw,kw. 773  18 ((exogenous or oral) adj3 creatine).tw,kw. 139  19 creatine intake.tw,kw. 16  20 (dietary creatine or creatine monohydrate).tw,kw. 330  21 or/15-20 912  22 14 and 21 70 | APA PsycInfo <1806 to September 2025 Week 5>  1 exp mental disorders/ 1144758  2 (anxiety or psychotic or psychosis or psychoses or schizo* or bipolar or manic or mania or delirious or depressi* or depresse* or major depression* or obsessive-compulsive or obsessive compulsive or compulsive obsessive or OCD or agoraphob* or panic or phobia or phobic or melanchol* or neurosis or neurotic or neuroses or conversion disorder*).tw. 739925  3 (alzheimer* or dementia or "substance use disorder*" or substance abuse or drug abuse or opiate abuse or opiate addiction or opioid abuse or opioid addiction or alcohol abuse or alcoholism or Dissociative disorder* or Elimination Disorder* or impulse control disorder* or disruptive behavio?r* or dissocial disorder* or personality disorder* or ptsd or paraphilic disorder* or factitious disorder*).tw. 312341  4 behavio?r disorder*.tw. 9087  5 (mental disorder* or mental* ill* or mood disorder* or Neurocognitive Disorder*).tw. 150350  6 (autism or autistic or attention deficit or adhd).tw. 111445  7 (eating disorder* or Anorexia or anorexic or bullimia or binge eating).tw. 44430  8 gambling.tw. 13219  9 post trauma*.tw. 23164  10 Catatoni*.tw. 3808  11 Catatonia/ 1703  12 neurodevelopment* disorder*.tw. 9002  13 (psychiat* adj2 (illness* or disorder*)).tw. 57634  14 or/1-13 1518043  15 (creatine adj5 loading).tw. 1  16 ((exogenous or oral) adj3 creatine).tw. 19  17 creatine intake.tw. 3  18 (dietary creatine or creatine monohydrate).tw. 51  19 (creatine and supplement*).mp. 183  20 or/15-19 207  21 14 and 20 71  22 (double-blind or random* asigned or control).tw. 556662  23 trial.tw. 137428  24 22 or 23 649547  25 21 and 24 23 |

eTable 3. List of studies excluded after full-text assessment.

| **Study** | **Reason for Exclusion** |
| --- | --- |
| Amital 2006[^1^](https://www.zotero.org/google-docs/?SnKRuN) | Wrong study design |
| Hellem 2015[^2^](https://www.zotero.org/google-docs/?8p6mD0) | Wrong study design |
| Kaptsan 2007[^3^](https://www.zotero.org/google-docs/?b1XjvM) | Wrong study design |
| Kious 2017[^4^](https://www.zotero.org/google-docs/?RDqTic) | Wrong study design |
| Kondo 2011[^5^](https://www.zotero.org/google-docs/?TPCGHz) | Wrong study design |
| Roitman 2007[^6^](https://www.zotero.org/google-docs/?RuK87h) | Wrong study design |
| Sung 2024[^7^](https://www.zotero.org/google-docs/?0e35u5) | No full text |
| Toniolo 2017[^8^](https://www.zotero.org/google-docs/?EXwuIP) | Wrong outcome |
| Toniolo 2017[^8^](https://www.zotero.org/google-docs/?Pfffs5) | Duplicate |
| Toniolo 2018[^9^](https://www.zotero.org/google-docs/?vJh0T0) | Duplicate |
| IRCT2015040521609N1[^10^](https://www.zotero.org/google-docs/?1qzYFu) | No full text |
| NCT00140192[^11^](https://www.zotero.org/google-docs/?mDluBF) | Wrong study design |
| NCT01601210[^12^](https://www.zotero.org/google-docs/?VrU17w) | Duplicate |
| NCT04504253[^13^](https://www.zotero.org/google-docs/?3FrEsK) | No full text |

**References**

[1. Amital D, Vishne T, Roitman S, Kotler M, Levine J. Open study of creatine monohydrate in treatment-resistant posttraumatic stress disorder. J Clin Psychiatry. 2006 May;67(5):836–7.](https://www.zotero.org/google-docs/?rklJzG)

[2. Hellem TL, Sung ,Young-Hoon, Shi ,Xian-Feng, Pett ,Marjorie A., Latendresse ,Gwen, Morgan ,Jubel, et al. Creatine as a Novel Treatment for Depression in Females Using Methamphetamine: A Pilot Study. Journal of Dual Diagnosis. 2015 Oct 2;11(3–4):189–202.](https://www.zotero.org/google-docs/?rklJzG)

[3. Kaptsan A, Odessky A, Osher Y, Levine J. Lack of efficacy of 5 grams daily of creatine in schizophrenia: a randomized, double-blind, placebo-controlled trial. J Clin Psychiatry. 2007 Jun;68(6):881–4.](https://www.zotero.org/google-docs/?rklJzG)

[4. Kious BM, Sabic H, Sung YH, Kondo DG, Renshaw P. An open-label pilot study of combined augmentation with creatine monohydrate and 5-hydroxytryptophan for SSRI- or SNRI-resistant depression in adult women. Journal of clinical psychopharmacology. 2017 Oct;37(5):578.](https://www.zotero.org/google-docs/?rklJzG)

[5. Kondo DG, Sung YH, Hellem TL, Fiedler KK, Shi X, Jeong EK, et al. Open-label adjunctive creatine for female adolescents with SSRI-resistant major depressive disorder: A 31-phosphorus magnetic resonance spectroscopy study. Journal of Affective Disorders. 2011 Dec 1;135(1):354–61.](https://www.zotero.org/google-docs/?rklJzG)

[6. Roitman S, Green T, Osher Y, Karni N, Levine J. Creatine monohydrate in resistant depression: a preliminary study. Bipolar Disorders. 2007;9(7):754–8.](https://www.zotero.org/google-docs/?rklJzG)

[7. Sung YH, Shi X, Nielson H, Kondo D, Renshaw P, Kious B. Brain Phosphorus Bioenergetics Improves in Depressed Subjects After Eight Weeks of Creatine Monohydrate Supplementation. Biological Psychiatry. 2024 May 15;95(10):S190.](https://www.zotero.org/google-docs/?rklJzG)

[8. Toniolo RA, Fernandes F de BF, Silva M, Dias R da S, Lafer B. Cognitive effects of creatine monohydrate adjunctive therapy in patients with bipolar depression: Results from a randomized, double-blind, placebo-controlled trial. Journal of Affective Disorders. 2017 Dec 15;224:69–75.](https://www.zotero.org/google-docs/?rklJzG)

[9. Toniolo RA, Silva M, Fernandes F de BF, Amaral JA de MS, Dias R da S, Lafer B. A randomized, double-blind, placebo-controlled, proof-of-concept trial of creatine monohydrate as adjunctive treatment for bipolar depression. J Neural Transm (Vienna). 2018;125(2):247–57.](https://www.zotero.org/google-docs/?rklJzG)

[10. The effect of Creatine adjunct on Athletes Mood and Affect. [Internet]. 2018 Feb [cited 2025 May 22]. Report No.: IRCT2015040521609N1. Available from: https://trial.medpath.com/clinical-trial/314a2a41cc567d33/irct2015040521609n1-creatine-effect-athletes-mood-body-building](https://www.zotero.org/google-docs/?rklJzG)

[11. Creatine as a New Treatment for Schizophrenia:A Double-Blind Trial [Internet]. clinicaltrials.gov; 2009 Jul [cited 2025 May 22]. Report No.: NCT00140192. Available from: https://clinicaltrials.gov/study/NCT00140192](https://www.zotero.org/google-docs/?rklJzG)

[12. Placebo-Controlled Dose-Ranging Trial of Creatine Augmentation for Adolescent Females With Treatment-Resistant Major Depressive Disorder: a Magnetic Resonance Spectroscopy Study [Internet]. clinicaltrials.gov; 2016 Oct [cited 2025 May 22]. Report No.: NCT01601210. Available from: https://clinicaltrials.gov/study/NCT01601210](https://www.zotero.org/google-docs/?rklJzG)

[13. A Pilot Study of Creatine Monohydrate as an Augmenting Agent for ECT in Persons With Major Depressive Disorder [Internet]. clinicaltrials.gov; 2024 Nov [cited 2025 May 22]. Report No.: NCT04504253. Available from: https://clinicaltrials.gov/study/NCT04504253](https://www.zotero.org/google-docs/?rklJzG)
